# Understanding Interaction Patterns within Deep-Sea Microbial Communities and Their Potential Applications

**DOI:** 10.3390/md20020108

**Published:** 2022-01-28

**Authors:** Muhammad Zohaib Nawaz, Raghul Subin Sasidharan, Huda Ahmed Alghamdi, Hongyue Dang

**Affiliations:** 1 State Key Laboratory of Marine Environmental Science, Fujian Key Laboratory of Marine Carbon Sequestration, College of Ocean and Earth Sciences, Xiamen University, Xiamen 361102, China; zohaib@xmu.edu.cn; 2 Department of Zoology, Government College Kariavattom, Thiruvananthapuram 695581, India; raghulzubin@gmail.com; 3 Department of Biology, College of Sciences, King Khalid University, Abha 61413, Saudi Arabia; dr.huda.gh@gmail.com

**Keywords:** microbial interactions, microbial community, community function, correlation network analysis, deep sea

## Abstract

Environmental microbes living in communities engage in complex interspecies interactions that are challenging to decipher. Nevertheless, the interactions provide the basis for shaping community structure and functioning, which is crucial for ecosystem service. In addition, microbial interactions facilitate specific adaptation and ecological evolution processes particularly essential for microbial communities dwelling in resource-limiting habitats, such as the deep oceans. Recent technological and knowledge advancements provide an opportunity for the study of interactions within complex microbial communities, such as those inhabiting deep-sea waters and sediments. The microbial interaction studies provide insights into developing new strategies for biotechnical applications. For example, cooperative microbial interactions drive the degradation of complex organic matter such as chitins and celluloses. Such microbiologically-driven biogeochemical processes stimulate creative designs in many applied sciences. Understanding the interaction processes and mechanisms provides the basis for the development of synthetic communities and consequently the achievement of specific community functions. Microbial community engineering has many application potentials, including the production of novel antibiotics, biofuels, and other valuable chemicals and biomaterials. It can also be developed into biotechniques for waste processing and environmental contaminant bioremediation. This review summarizes our current understanding of the microbial interaction mechanisms and emerging techniques for inferring interactions in deep-sea microbial communities, aiding in future biotechnological and therapeutic applications.

## 1. Background

Considerable studies exploring microbial interactions at the community level have been done during the last decades [1,2,3,4,5]. Generally, the microorganisms living in a specific community may cooperate or compete for nutrients and other resources. They may exchange signal molecules and metabolites as well. Such interactions provide innate mechanisms in shaping the community structure, ecological function, and temporospatial dynamics of the microbiomes observed in various environments [6,7,8]. Intra- and interspecific interactions lay the foundation of the so-called “microbial community intelligence”, which can be explored for a variety of applications [9]. An interaction may exert a positive (win), negative (lose), or neutral (zero) impact on the individual microorganisms involved in the specific inter-species interaction [10,11,12]. Depending on the outcomes of the interaction between interacting species, the interaction can be classified into one of several distinct situations [13], such as a win-win (mutualism) [14,15,16,17], win-lose (parasitism, predation) [18,19], win-zero (commensalism) [7,20], lose-lose (competition) [21], or a zero-lose (amensalism) relationship [10,22,23]. Although microbial interactions play important roles in driving the ocean’s biogeochemical cycles [24] and the formation of coupled (or decoupled) community taxon-function dynamics in ecosystems [25], exploring the various types of interactions among the microorganisms in a complex community is not straightforward. Furthermore, interspecies interactions in microbial communities are not static, and evolution in interspecies interactions may occur over ecological timescales [26]. The interaction of evolution and ecology adds another layer of complexity to microbial interactions. Although it is a challenge to decode, the evolvability of microbial interactions contributes to the ecosystems’ ecological memory and adaptive capacity, which may play critical roles in enabling the ecosystems to prepare for, and respond to, future perturbations such as the impacts of global change [27].

**Figure 1:**
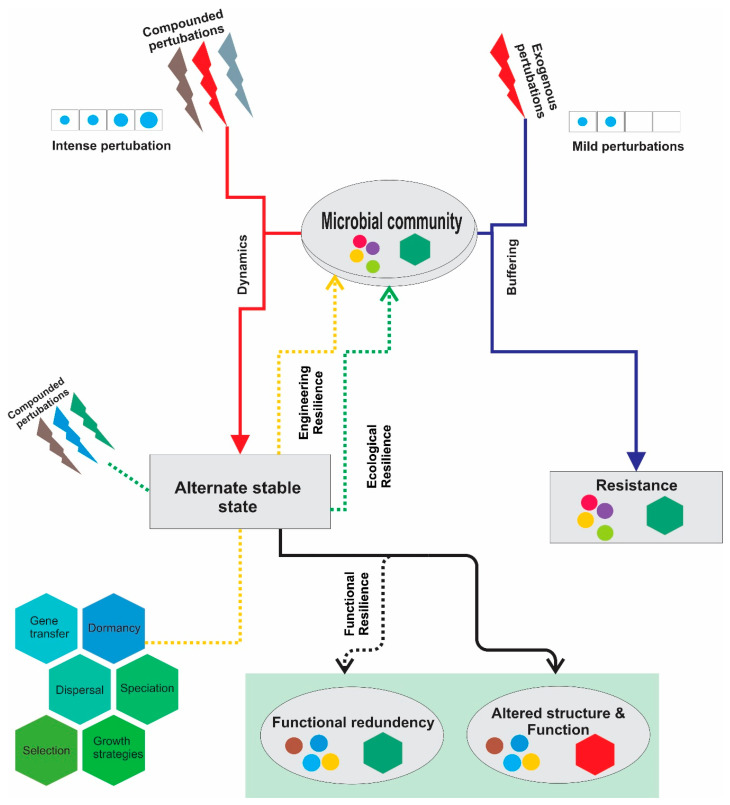
Effect of environmental disturbances on the microbial community composition.

Recent time-series analyses have shown that some microbial communities may change in a resilient manner in response to environmental change (ecological resilience) [28], with the system attaining its original structure with time (engineering resilience) in response to species actions, including selection, dormancy, and speciation [29]. Species interactions may support bringing back the community’s steady state in response to environmental perturbations (Figure 1) (e.g., changes in temperature, pH, oxygen concentration, redox potential, and nutrient supply) that trigger a change in the community structure [30]. Alternatively, some other microbial communities do not necessarily show resilience. Instead, they may tend to achieve an alternate stable state after a change in the environment [31]. Functional redundancy among distinct microbial species may provide a mechanism to maintain the community functionality with varied community compositions [32,33,34]. The effect of environmental perturbations on the microbial community structure has been illustrated in Figure 1. Microbiota in different environments may harbor varied taxonomic compositions. Nevertheless, they may host highly conserved community gene content and thus similar functional potentials [35]. Therefore, microbial communities can display one (mono-), two (bi-), or more (multi-) stable states under the same environmental conditions. The existence of a stable state(s) makes the microbial communities somehow fathomable [30,36]. It is possible to predict complex and dynamic interactions even within microbial communities in deep oceans [37].

The growing availability of microbial data in the marine environment reveals that the microbial interactions among species are more complex than previously thought. Emerging tools are being developed to infer such complex interactions. This review summarizes existing knowledge of the microbial interaction mechanisms and research tools for inferring relationships in deep-sea microbial communities, aiding future biotechnological and therapeutic applications.

## 2. The Complexity of Microbial Interaction in the Deep-Sea Environment

The deep-sea environments constitute vast and variable habitats for microorganisms, including viruses, archaea, bacteria, fungi, and protists. The deep-sea microorganisms usually form complex ecological interaction webs instead of dwelling in isolation. They are the key players in the deep-sea biogeochemical cycling of bio-essential elements, such as carbon, nitrogen, phosphorus, sulfur, and various trace metals [38,39]. A large number of microorganisms dwell in energy-deficient deep ocean sediments, which are considered the largest ecosystem on Earth [40]. Moreover, deep-sea hydrothermal vent chimneys characterized by steep physicochemical gradients harbor unique microbial communities that are particularly enriched with chemolithoautotrophic bacteria and archaea [41]. Similarly, the deep-sea cold seeps also harbor one of the most productive ecosystems in the ocean, supporting complex microbial interactions centered on the coupling of anaerobic methane oxidation and sulfate reduction [42,43,44]. Due to the great demand for nitrogenous nutrients by the cold seep chemosynthetic ecosystems [45], nitrogen fixation by anaerobic methane-oxidizing archaea provides a critical mechanism to cope with the in-situ nitrogen deficiency [46,47,48]. Microbial interactions form the primary force driving the coupled cycling of carbon, nitrogen, sulfur, and other bio-essential elements in both hydrothermal vent and methane seep environments.

The subseafloor deep biosphere is another extreme environment of the ocean. Due to the lack of sunlight and the extremely scarce supplies of organic matter from the surface ocean, the growth and eco-physiological activities of microorganisms living in the deep biosphere are highly limited by the meager availability of energy and organic substrates [49,50,51]. Under such resource-limited conditions, interspecies interactions such as metabolite cross-feeding and biosynthetic complementation may play a critical role for the in- situ microbial communities to fully exploit the available energy and growth substrates. Microorganisms carry out biochemically catalyzed redox reactions for energy transduction in the deep biosphere, where metabolically usable electron donors include methane, hydrogen, reduced iron, reduced manganese, reduced sulfur, ammonia, and ammonium. The electron acceptors in the deep biosphere include oxygen, oxidized nitrogen compounds such as nitrate and nitrite, manganese and iron oxides, oxidized sulfur compounds such as sulfate and sulfite, and oxidized carbon compounds such as carbon dioxide [52,53]. Different electron donors and acceptors are available in distinct habitats of the deep biosphere, forming the primary driving force to shape the community diversity, ecological function, and biogeography of the microorganisms inhabiting therein [51,54]. For example, in the oxic layer of the deep-sea sediments, aerobic microorganisms such as ammonia-oxidizing archaea and bacteria and nitrite-oxidizing bacteria may be the major chemolithoautotrophs contributing to inorganic carbon fixation [55]. In contrast, chemolithoautotrophic anaerobes contribute to the in-situ dark carbon fixation in the deep anoxic layers of sediments. The subseafloor deep biosphere also represents other extreme conditions such as extreme temperature and high pressure. Despite improved knowledge of the microbial existence and diversity in the deep biosphere, mechanisms regarding habitat adaptation, metabolic activities, and interspecific interactions of the in-situ microbial communities remain largely elusive [56,57,58].

Studies have revealed through various molecular techniques not only the astonishing diversity but also the temporospatial dynamics of microbial abundance in deep-sea environments [59,60]. The microbial communities have usually been studied through phylogenetic analyses using taxonomic biomarkers such as 16S and 18S rRNA gene sequences [61,62]. Although these methods have made substantial contributions to the advancement of microbial ecology, they have certain limitations, including lineage missing caused by PCR primer mismatches and the inability of using single marker gene-based data to decode metabolic pathways and interactions in a microbial community [63]. Fortunately, these limitations have been overcome recently using multiple-omic analyses [64,65,66,67]. The deep-sea environments contain a vast diversity of microbial species and physiological traits [68], providing an opportunity for understanding microbial interactions in such ecologically and climatically critical earth subsystems.

Marine sediments contain a massive reservoir of living microorganisms, most of which may attach to sediment particles and live in biofilms thereon [69,70]. This surface-associated lifestyle may prompt various interactions among the sediment-dwelling microorganisms [5]. Microbes residing in biofilms are metabolically and functionally integrated microbial communities, displaying a high degree of organization and functioning as a unit with shared metabolites and signaling compounds [71]. Biofilms also facilitate gene expression regulation and horizontal gene transfer among community members [5]. Collective behavior of the microbial community is established by microbial interactions, such as those via the quorum sensing (QS)-based cell-to-cell communication mechanisms that allow the interacting microbes to share information, materials, and functions [72]. QS communication is a response to microbial density that relies on the exchange of extracellular signaling molecules called autoinducers. QS enables microbial communities to behave like multicellular organisms, displaying mutual benefit, altruism, selfishness, or other social traits [73]. Microbes also possess other cell-to-cell communication mechanisms, such as the vesicle-mediated signal molecule transportation system [74] and the intercellular nanotubes-based microbial communication network [75]. Intra- and inter-species communications enable microbial communities to coordinate various ecophysiological processes, such as symbiosis, virulence, antibiotic production, and biofilm formation [76]. Engaging in social activities also enhances the survival of participating microbes in highly complex or adverse environments [71,77].

The interspecific interactions in sediment microbial communities have seldom been studied, particularly at the microscales that may provide the most relevant biological and ecological information about the in-situ microbial processes and mechanisms. The sediment microorganisms are essentially the engine driving the cycling of carbon and nutrients in the marine benthic ecosystems. They are also the cell factory carrying out biodegradation, biotransformation, and bioremediation of various contaminants and pollutants entering the ocean [78,79]. Marine sediments provide specific physicochemical and nutrient gradients, facilitating complex microbial communities and interspecific interactions. Seawater particles may provide similarly complex microenvironments, facilitating microbial interactions in the otherwise seemingly homogeneous bulk seawater environments [5]. The microbial interactions associated with seawater particulate organic matter play an important role in mediating the carbon sequestration efficiency of the biological carbon pump, a central mechanism played by the marine ecosystems for regulating the atmospheric CO_2_ concentration and thus the climate [80]. Intensified microbial interactions associated with seawater particles and marine sediments may help the in-situ microbial communities evolve novel metabolic pathways and chemical compounds, presenting the potentials in biomedical, biotechnological, and industrial applications [81]. In highly challenging habitats such as the deep ocean environments, microorganisms existing as interacting community members may foster the ability to perform complex metabolic tasks via communicational cooperation and division of labor [82]. These ecological principles can be applied to the design and implementation of synthetic microbial communities for specific biomolecules and functions, revolutionizing the application of microbes and their interactions for therapeutic and biotechnological purposes [82,83].

**Figure 2:**
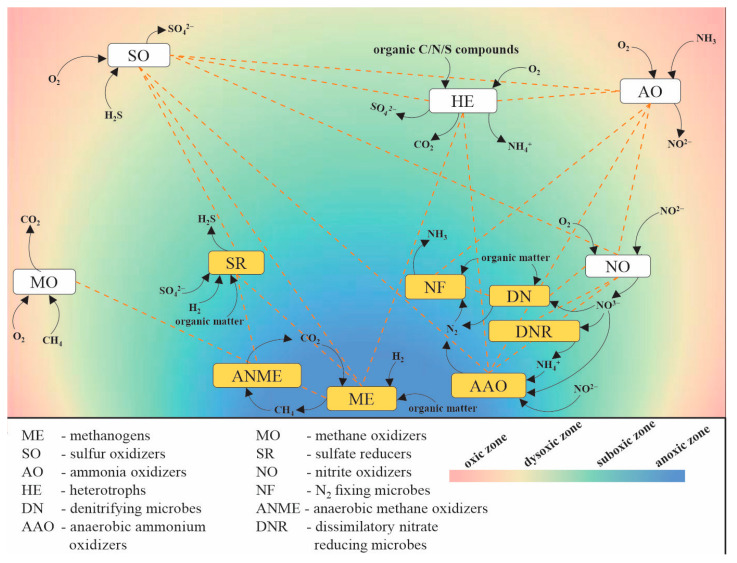
Interaction among C, N, and S cycling microbes associated with seawater particle aggregates in the deep-sea oxygenated waters.

Seawater sinking particles, such as macroscopic “marine snow” aggregates, act as a vector for transporting surface ocean-derived organic matter to the deep waters and seafloor [84]. Thus, they provide substantial organic substrates and nutrients to the deep ocean ecosystems. In addition, marine particles provide unique and partially isolated microenvironments by creating micro-scale suboxic or even anoxic conditions in the otherwise oxygenated bulk seawater of the ocean [5,85]. Diverse and interacting aerobic and anaerobic microbes colonize different niches of marine particles, taking advantage of the various organic and inorganic chemicals as growth substrates and electron donors and acceptors for energy metabolism (Figure 2). The productivity of the deep oceans may be highly influenced by the interaction of the particle-associated microbes, which form complex networks mainly characterized by substrate-level interdependencies. The heterotrophs seen in the aerobic and suboxic microzones of the marine particles (Figure 2) perform degradation of organic biopolymers, simultaneously consuming oxygen to maintain the redox gradient in the particles [5,55]. The abundances of particle-associated heterotrophs are usually orders of magnitude higher than those living in the surrounding seawater [86]. Microzonal oxygen-deficient conditions in the core of the marine snow particles facilitate anaerobic processes such as microbial denitrification, sulfate reduction, and methane production. The occurrence of ammonia and nitrite oxidizers in the oxic and suboxic microzones coupled with anaerobic ammonium oxidizers and denitrifiers in the anoxic microzones of the marine particles suggests an effective mechanism for the loss of fixed nitrogen in the ocean [87]. Similarly, coupled cycling of carbon, nitrogen, and sulfur was suggested in particular oxycline microzones of marine snow particles [85], where electron donors and acceptors may be actively recycled between distinct oxidation states (Figure 2). This network of biogeochemical interdependencies suggests the pivotal role of particle-associated microbes in the ecosystem metabolism of the deep ocean.

## 3. Advancements in Molecular Techniques for Exploring Species Interaction

Species interactions in deep-sea microbial communities can be inferred either by analyzing their taxonomic data from different sampling sites at a given time or by analyzing time-series samples from the same sampling site. Moreover, an inferred interspecific relationship can be validated by investigating samples collected further from more habitats or from longer timescales [88,89]. As the availability of required samples from different environmental sites is usually not feasible, the time-series approach is more commonly opted for inferring species interactions. In this regard, ocean time-series study sites, such as the Bermuda Atlantic Time-series Study (BATS) site in the Sargasso Sea [90], the Hawaii ocean time-series (HOT) program Station ALOHA [91], and the South-East Asian Time-series Study (SEATS) site in the South China Sea [92], may prove valuable in providing ecologically meaningful materials and information for deep-sea microbial interaction analyses. The availability of time-series analysis tools has made it possible to develop predictive models and construct time-varying networks [93].

Experimental approaches for discovering interactions between species are mainly based on the concept of Gause’s co-culture experiments [94], in which species interactions are tested by developing an artificial community in a controlled environment [95,96]. Although classical co-culture experiments can answer many ecological questions, they can only help reveal interactions among a limited number of species. Considerable progress has been made recently to extend the applications of co-culture experiments to include complex communities. However, there are still challenges, including identifying certain microbial community members, particularly those microbes that are difficult or even impossible to obtain pure strains.

Advancements in molecular technologies have made it possible to study complex microbial communities. For example, the development of PhyloChip and GeoChip for high-throughput co-culturing experiments may help reveal species interactions, even in deep-sea microbial communities [97]. Isotope labeling and probing experiments are helpful in deciphering the flow of metabolites and metabolic connections in microbial consortia [98]. Fluorescence in-situ hybridization (FISH)-based methods are helpful to explore the interaction pattern of co-aggregated species [98,99]. In combination with the microautoradiography (MAR) technique, the FISH technique holds the potential for observing the incorporation of radioisotopes in interacting microbial cells [100]. Similarly, the combination of FISH with Raman microscopy or high-resolution nanometer-scale secondary-ion mass spectrometry (NanoSIMS) can reveal microbial interactions with the use of stable isotope-labeled substrates [101,102,103].

The Deep-Sea Drilling Project made the first effort to collect and study microbial samples from various depths of marine sediments. Ever since these pioneering studies, growing molecular technologies have been used to study species interactions in deep-sea ecosystems. The first application of the combined FISH and secondary-ion mass spectrometry (SIMS) technique for exploring microbial interactions was reported by Orphan et al. [104], who studied methane-rich deep-sea sediments to determine the role of archaeal and bacterial cells in the anaerobic oxidation of methane. This study revealed the physical association of anaerobic methanotrophic archaea (ANME) with sulfate-reducing bacteria (SRB), suggesting that the interacting ANME and SRB consortia are responsible for the observed methanotrophy in anoxic cold seep sediments. Nowadays, next-generation “omics” approaches, including metagenomics, metatranscriptomics, metaproteomics, and metabolomics, have been being developed. These techniques hold great potential for the decoding of species interactions in deep-sea microbial communities [99,105].

## 4. Approaches for Exploring Species Interactions

### 4.1. Inferring Microbial Interactions through Co-Occurrence Pattern Analyses

Different approaches for exploring interspecific interactions in microbial communities have been developed, including co-occurrence pattern analyses, community metabolic pathway inferring, and eco-energetic modeling [13,106,107,108]. Co-occurring species may have similar ecological characteristics or may associate with each other because of physiological interdependencies or fine-scale niche differentiation. Thus, co-occurrence patterns may provide important insights into the temporospatial and functional distribution of microbes and the environmental complexity within an ecosystem. Microbial species with similar ecological traits can hardly co-exist in an environment when their common resources become limited. Competitive exclusion under resource-limiting conditions may eliminate some of the competing species that depend on the same limiting substrate for growth or survival [109]. However, species co-existence may stem from many distinct mechanisms, confounding the ecological explanation of an observed co-occurrence pattern. For example, Leinweber et al. recently proposed a “cheating effect” mechanism for fostering the co-existence of competitive species in a microbial community [110]. Under resource-limiting conditions, intraspecific competition caused by bacterial cheaters may alleviate interspecific competition, thus fostering species co-existence in a microbial community [110].

Co-occurrence patterns may be sensitive to different spatial scales or ecosystems. Williams et al. compared the co-occurrence data taken from different ecosystems, finding that only a few co-occurring pairs of microbial species showed consistency across different ecosystems. Most of the co-occurrence relationships detected in individual ecosystems were inconsistent across different ecosystems [111]. These results highlight the instability of using co-occurrence pattern analyses in inferring microbial interactions. Interactions among microbes are rooted in metabolic connections, providing a mechanistic and thus more reliable approach for detecting interacting microbes.

### 4.2. Inferring Microbial Interactions through Community Metabolic Pathway Analyses

The deep-sea ecosystem is the largest and most challenging ecosystem on Earth [112,113]. Most parts of this system are resource-limited. Recycling and reusing growth substrates and energy materials facilitate the maintenance and functioning of the deep-sea ecosystem, where the microorganisms may heavily rely on metabolite sharing to complement each other’s biosynthetic requirements. Understanding metabolic exchanges and other forms of metabolic cooperation holds the key to decoding the microbial interactions therein. Community metabolic pathway analyses are challenging, particularly for the remote deep-sea microbial communities. Integrated utilization of diverse technologies, from metagenomics to isotope tracing, should facilitate the detection of metabolic pathways and networks in the deep-sea microbial communities [114].

**Figure 3:**
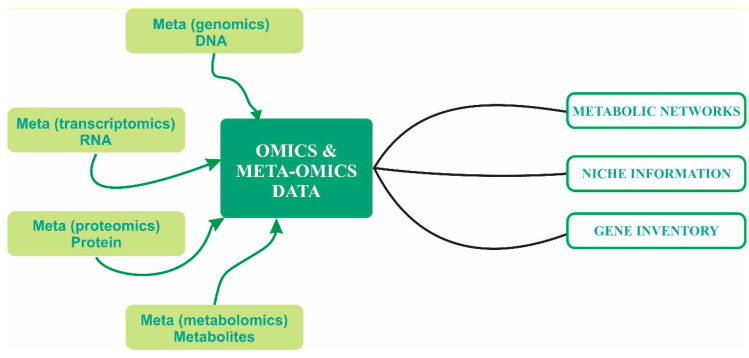
Meta-omics approaches for studying microbial communities and their functions.

Meta-omics techniques are powerful tools for identifying species and metabolic potentials in a microbial community and inferring interactions among different community members. Data generated by these techniques can be used to formulate and test new hypotheses about microbial metabolic interactions (Figure 3). A microbial community as a whole can be treated as a super- or mega-organism, whose metabolic pathways and networks are fathomable with the use of the -omics approaches [115,116]. For example, cooperation or competition among microbes may be inferred taxonomically from time-series analysis of a specific community [117]. Meta-omics analyses with emphases on the microbial functional traits may be applied subsequently to corroborate further or contradict the inference made by taxonomy-based analyses [13,118,119]. Metagenomics data usually reveal the metabolic potentials of a microbial community, while metatranscriptomics and metaproteomics data may reveal more about the metabolic activities of the studied microbial community [120,121]. The molecular sequence-based –omics techniques can be further combined with isotope tracing techniques to explore the flow of metabolites in a microbial community [122]. Advanced microscopy imaging techniques, such as cryogenic transmission electron microscopy, have been developed to visualize physical interactions among microbial cells in complex microbial communities [123]. Imaging mass spectrometry has also been developed to visualize interspecies metabolic exchange between interacting microbial cells [124,125]. These cutting-edge techniques will undoubtedly produce more exciting discoveries in deep-sea microbial interaction studies.

**Figure 4:**
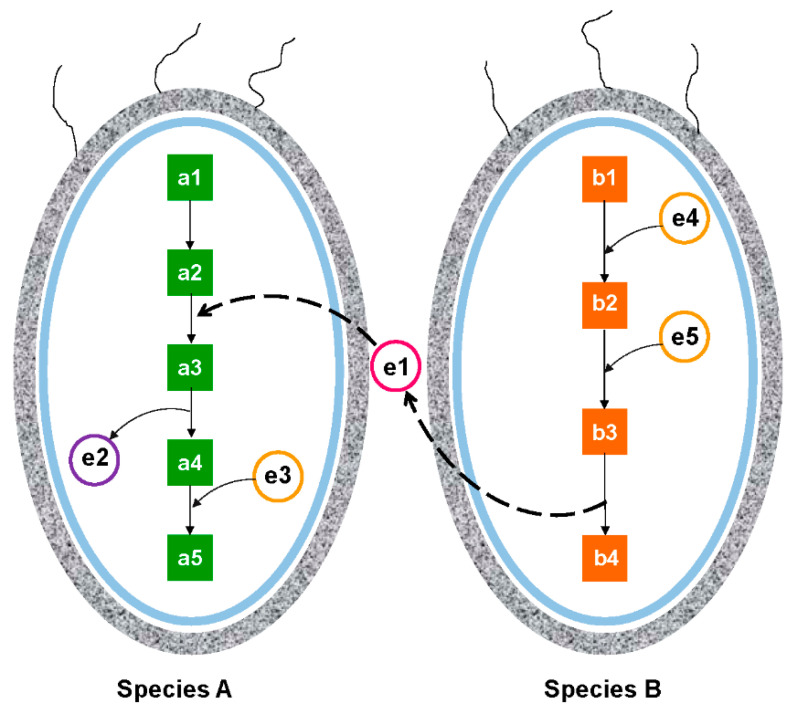
Two species in a microbial community interact with each other by sharing metabolites to fuel each other’s metabolic pathways. Metabolic pathway of species A needs metabolite “e1” that is not synthesized by its own metabolic machinery and needs to be taken up from species B through the environment. Therefore, species A must coexist with species B that synthesizes this metabolite. Components a1, a2, a3, a4, and a5 represent the genes in the metabolic pathway of species A, whereas b1, b2, b3, and b4 are genes in the metabolic pathway of species B. Components e1, e2, e3, e4, and e5 are different intermediate metabolites produced or needed by the two species.

Advancements in molecular techniques, particularly the—omics ones, have improved our understanding of microbial ecology, providing the essential data and concepts for mathematical modeling to predict metabolic interactions of the various microbial communities [126,127,128]. Sequenced genomes facilitate the construction of the metabolic network in single organisms by providing the necessary information of the metabolic enzymes and the biochemical reactions they catalyze [129]. Community metabolic networks can be constructed using the microbial community’s metagenomic data [130]. From a constructed metabolic network, it is convenient to identify the metabolites that are not synthesized by a microorganism but may be obtained from other community members [131,132]. Potential substrate competitions among different microorganisms can also be identified in a community metabolic network. Therefore, community metabolic networks provide critical information about the species-specific resource requirements and metabolic cooperation and competition in a given microbial community [130,133], providing valuable insights into the processes and mechanisms of microbial interactions (Figure 4).

Metabolic networks also provide information about the community’s metabolic environment [134]. The habitat conditions and corresponding microbial adaptation mechanisms may be inferred from metabolic network details [131,132,135,136]. Valuable information about the habitat attributes and microbial adaptation strategies may be used to design culture media to isolate interested microorganisms.

### 4.3. Inferring Microbial Interactions through Community Eco-Energetic Modeling

Microbes dwelling the deep-sea environments conserve energy via various redox reactions. The coupling of oxidation and reduction reactions between different microbes facilitates microbial interactions along various energetic substrate gradients [55,137,138]. For example, marine sediments are rich in metal oxides and other minerals that serve as electron donors or electron acceptors in many microbiologically-catalyzed redox reactions [139,140]. Although the microbial cell envelope forms a permeability barrier to minerals, many microbes have evolved extracellular electron transfer mechanisms for using minerals to exchange electrons [140,141,142,143]. Marine mineral-mediated redox reactions thus can directly or indirectly facilitate microbial interspecific electron transfer and thus microbial interactions [144]. Redox coupling without mineral involvement also prevails in natural microbial communities. For example, aggregates or other forms of consortia formed by different microbes are common in marine environments. They facilitate otherwise difficult microbial metabolism, such as anaerobic oxidation of methane (AOM) [145,146]. Facilitated interspecific electron transfer between methane-oxidizing archaea and sulfate-reducing bacteria has been proposed as the primary mechanism for AOM in the aggregates [147,148].

Different energetic substrates (i.e., electron donors and electron acceptors) exist in distinct marine environments. The specific pairing of the available electron donors and their counterpart electron acceptors dictates the eco-energetic processes and the functional groups of microbes and the interaction pattern that can occur in a given environment [55,149,150]. Here, we propose that understanding the electron transfer interactions between different microbial species would help reveal some major interaction mechanisms and environmental adaptation strategies of deep-sea microorganisms. Combining the -omics techniques and eco-energetic modeling may prove fruitful to achieve this aim.

### 4.4. Synthetic Microbial Communities in Biotechnological and Therapeutic Applications

Interspecific interactions enable microbes to survive in highly challenging environments. Metabolic cooperation helps the deep-sea microbes maximize utilizing the available resources, including recalcitrant organic substrates [151], such as cellulose, lignin, chitin, lipids, and hydrocarbons. For example, syntrophic interactions between Lokiarchaeota and nitrite- or sulfite-reducing bacteria may enable these archaea to anaerobically degrade aliphatic and aromatic hydrocarbons in marine subsurface sediments [152]. On the contrary, acetogenic Bathyarchaeota may help fuel the marine subsurface ecosystem by providing organic substrates for heterotrophy and acetoclastic methanogenesis [40].

The capability of deep-sea microbes for collaborative degradation of complex and recalcitrant organic compounds may have many applications, such as biofuel production from cellulose, lignin, and other polysaccharide substances. The organic-decomposing communities and their enzymes are also useful for developing bioremediation techniques for coping with various environmental contaminants [153]. In addition, metagenomics-based approaches also reveal a plethora of enzymes that may have other potential applications, such as in the energy, biomedical, industrial, and biotechnological fields [154]. Enzymes from extremophiles (e.g., thermophiles and psychrophiles) deserve special attention because extremozymes’ higher stability and catalytic efficiency may have many therapeutic and biotechnological potentials [155,156].

Opportunistic pathogens usually lead to polymicrobial infections. An improved understanding of their colonization mechanisms and cell-to-cell communications is necessary for developing effective therapeutic strategies for diseases caused by multispecies infections [157]. The microbial QS-based cell-to-cell communication system is a promising target for interfering microbial interactions to prevent pathogen colonization, biofilm formation, and polymicrobial infections [82,158]. Some marine sediment bacteria secrete AHL-lactonase enzymes that can disrupt QS-mediated microbial interactions [159,160]. The QS-disrupting enzymes and the microbes producing them hold promising potential for tackling biofilm formation and infections by bacterial pathogens. A practical and potentially effective strategy is to engineer microbial symbioses involving AHL-lactonase-producing microbes to control bacterial infections [161]. Engineered microbial symbionts may also be applied to invade and kill cancerous cells [162]. Furthermore, autoinducer antagonists such as QS inhibitors may be developed and employed as effective antimicrobial drugs [163]. The deep-sea microorganisms may harbor many novel bio-chemicals and mechanisms that can interfere with microbial interactions, potentially appliable in therapeutics [164].

## 5. Future Directions

Species interactions are an important force shaping community structure and function. Species interactions can also strongly impact how organisms and their communities respond to global warming, ocean acidification, ocean deoxygenation, and other environmental stressors [165]. Inferring species interactions of the deep-sea microbial communities is still a challenging task. However, technological integrations, particularly those involving combined –omics, isotope tracing, and modeling approaches, make high-quality research possible in this field. New concepts and innovative theories may be further developed to achieve an ecosystems biology vision of the complex marine microbial communities [166,167]. Understanding the microbial interactions provides a gateway to the design of synthetic microbial communities to obtain novel or unique community functions.

**Figure 5:**
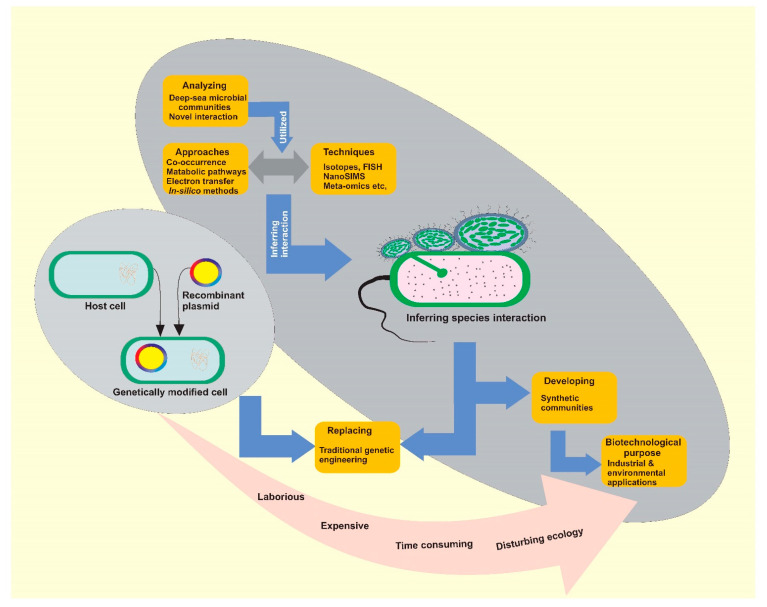
Advantages of synthetic communities’ applications over traditional genetic engineering at industrial scale.

Deep-sea microbial interaction research has been entering a golden age of rapid development since the turn of the new millennium. It is reasonable to predict that new scientific discoveries and theoretical advancements will thrive, which may help advance the applications of interspecific interaction-based biotechniques in many fields, such as therapeutics and bioremediation. For example, the use of synthetic bioremediation communities at the industrial scale has several advantages over the traditional genetic engineering approaches (Figure 5), such as greater adaptability in extreme conditions and lesser adverse effect on the ecology of the in situ microbial communities. Exploring the interactions in the deep-sea microbial communities opens new horizons for the advancement of microbial ecology and the applications of microbial theories for therapeutic and biotechnological purposes.
